# Size dependent heat generation of magnetite nanoparticles under AC magnetic field for cancer therapy

**DOI:** 10.1186/1477-044X-6-4

**Published:** 2008-10-20

**Authors:** Jun Motoyama, Toshiyuki Hakata, Ryuji Kato, Noriyuki Yamashita, Tomio Morino, Takeshi Kobayashi, Hiroyuki Honda

**Affiliations:** 1 Nanotherapy Co., Ltd, 19-11, Kikui 2-chome, Nishi-ku, Nagoya 451-0044, Japan; 2 Department of Biotechnology, School of Engineering, Nagoya University, Furo-cho, Chikusa-ku, Nagoya 464-8603, Japan; 3 Toda Kogyo Corp., 1-4 Meijishinkai, Otake, Hiroshima 739-0652, Japan; 4 Department of Biological Chemistry, School of Bioscience and Biotechnology, Chubu University, 1200 Matumoto-cho, Kasugai, Aichi 487-8501, Japan

## Abstract

**Background:**

We have developed magnetic cationic liposomes (MCLs) that contained magnetic nanoparticles as heating mediator for applying them to local hyperthermia. The heating performance of the MCLs is significantly affected by the property of the incorporated magnetite nanoparticles. We estimated heating capacity of magnetite nanoparticles by measuring its specific absorption rate (SAR) against irradiation of the alternating magnetic field (AMF).

**Method:**

Magnetite nanoparticles which have various specific-surface-area (SSA) are dispersed in the sample tubes, subjected to various AMF and studied SAR.

**Result:**

Heat generation of magnetite particles under variable AMF conditions was summarized by the SSA. There were two maximum SAR values locally between 12 m^2^/g to 190 m^2^/g of the SSA in all ranges of applied AMF frequency and those values increased followed by the intensity of AMF power. One of the maximum values was observed at approximately 90 m^2^/g of the SSA particles and the other was observed at approximately 120 m^2^/g of the SSA particles. A boundary value of the SAR for heat generation was observed around 110 m^2^/g of SSA particles and the effects of the AMF power were different on both hand. Smaller SSA particles showed strong correlation of the SAR value to the intensity of the AMF power though larger SSA particles showed weaker correlation.

**Conclusion:**

Those results suggest that two maximum SAR value stand for the heating mechanism of magnetite nanoparticles represented by hysteresis loss and relaxation loss.

## Background

Hyperthermic cancer treatments have been used for many years, particularly in anticancer therapy [1]. However, efficiency of the treatment did not satisfy in the clinical scene, because of its difficulty of raising the objective tissue temperature properly [2]. There Magnetic Fluid Hyperthermia (MFH), by using the magnetite (Fe_3_O_4_) as a preferable heating source, due to its strong magnetic property and low toxicity, is a promising approach for treating cancer [3]. MFH can raise the temperature in the tumor locally up to 41–46°C if magnetic fluid was selectively introduced and therefore kill tumor cells directory without damages of ambient healthy cells. In this technique, magnetite particles that have ferromagnetic or superparamagnetic property are dispersed into the aqueous phase and introduced into tumor cells. In our previous study, magnetite nanopaticles covered with the cationic liposome (magnetite cationic liposomes, MCLs) to show higher adhesion properties to the cell surfaces that is charged negatively [4-6]. In previous animal studies, we have demonstrated the efficacy of hyperthermia induce using MCLs in several types of tumor model; for instance, B16 melanoma in mice [7,8], T9 glioma in rats [6,9], osteosarcoma in hamsters [10], prostate cancer in mice [11] and MM46 mouse mammary carcinoma [12]. magnetite cationic liposomes (MCLs) Introduced magnetite particles transform the energy of the AC magnetic field into heat by several physical mechanisms, and its efficacy strongly depends on the frequency of the outer field as well as the particle's magnetic properties correlated to its diameter [3,13].

In our present study, we drew attention to the specific-surface area (SSA) as an represented mediator for expressing particle size and microscopic structure. The SARs of those magnetic particles were studied under several conditions of AC magnetic field or strength of the power and the frequency were changed. Here, SAR is defined as the energy amount converted into heat per unit time and unit mass.

## Methods

### Materials

Magnetite nanoparticles with different diameters (defined by SSA and confirmed by TEM observation) were purchased from Toda Kogyo Co. (Hiroshima, Japan). The SSA of each samples were determined by BET method. Magnetic properties of those samples were also measured. Table 1 shows a list of magnetite nanoparticles used in the present paper. The shapes of all the magnetite samples were determined as beads like particles by TEM method. Average diameter and polydispersity index of the magnetite nanoparticles were also measured by the DLS method after dispersed into distilled water. Saturated magnetization and coercivity was measured by vibrating sample magnetometer (VSM-5, Toei Industry Co. Ltd., Tokyo, Japan).

**Table 1:** Physical properties of the magnetite particles for the experiments

	Particle diameter(nm)					
Sample	TEM method (nm)	DLS method		Saturated magnetization (Am^2^/kg)	Coercivity (k A/m)	SSA (m^2^/g)
		Diameter (nm)	Polydispersity Index			
A	120	1986	0.57	82.5	7.2	12
B	40	1657	0.45	75.0	10.4	30
C	14	539	0.28	67.7	6.4	57
D	11	109	0.15	63.2	3.0	74
E	11	109	0.21	64.1	2.3	84
F	10	109	0.25	57.9	1.2	92
G	10	146	0.23	57.5	6.0	107
H	10	93	0.30	51.6	3.0	121
I	10	84	0.18	52.9	0.32	125
J	10	94	0.29	49.7	1.4	131
K	10	107	0.19	48.9	3.5	145
L	10	130	0.25	47.4	2.6	159
M	10	105	0.22	38.1	0.9	190

Kappa-carrageenan and magnesium chloride were purchased from Wako chemicals (Osaka, Japan).

### Preparation of the heating samples

Magnetite nanopartiles were dispersed in distilled water with the same concentration of 20 mg/ml and treated with ultrasound sonication for 30 minutes. Those magnetite dispersions were filled in the cylindrical polypropylene tubes that has 15 mm inner diameter with the kappa-carrageenan and the dispersions were gelated by appropriated dose of magnesium chloride. Temperature increase was caused by the upwardly generated AC magnetic field from the surface of the irradiation coil of the AMF radiator. As a whole, 2 g of the samples that contained 5 mg of magnetite nanoparticles were put in the sample tubes. As an control, the kappa-carrageenan solution was put into the sample tube without magnetite dispersions, and gelated by magnesium chloride.

### Heating experiments

In the present study, the alternating-magnetic-field (AMF) generator incorporating a solenoid with a ferrite core (FC) was used [11]. Magnetic field was arranged to change its frequency and amplitude. A list of those arrangements of the experimental courses is shown in Table 2.

**Table 2:** Variation of the frequency and the electrical power for AMF irradiator

Applied frequency	Applied power (kW)
360 kHz	1.6, 3.5, 5.2
200 kHz	2.5, 6.0, 13.0
110 kHz	2.5, 6.0, 13.1

**Figure 1:**
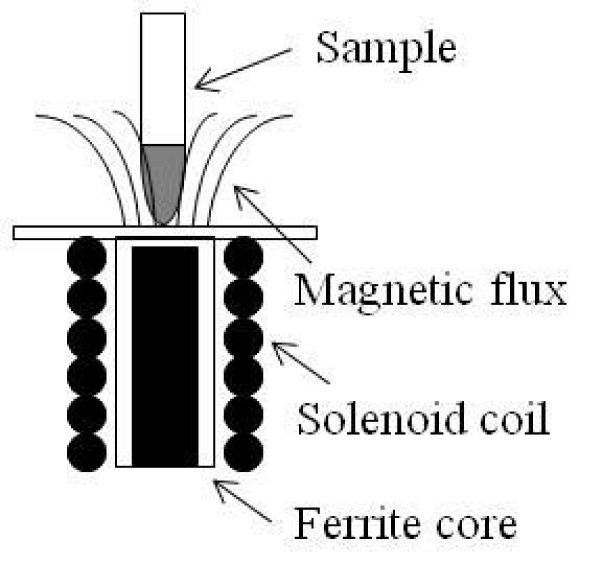
Illustration of experimental apparatus for SAR measurement.

All samples which were fleshly prepared for the present study were put on the irradiation coil as shown schematically in Fig. 1. Determination of magnetic field intensity over the ferrite core of our apparatus under AC field is very difficult. Therefore, the magnetic field intensity under DC field is represented. Indeed, the intensity under DC field was measured to be 32.5 kA/m at 13.0 W and it will be proportional to the power.

The temperature of the samples were measured by using optical thermometer (FX-9020; Anritsu Meter Co., Tokyo). After the experiments, all samples were dissolved by adding 12N hydrochloric acid and diluted to measure the iron contents by the potassium thiocyanate method [14].

Cnsequently we determined the magnetite concentration in the sample tubes precisely. The control was also exposed under the AMF, and confirmed that has no affection to heat generation. The SAR values of the samples were determined from the time dependent calorimetric measurements.

## Results

### Physical and magnetic properties of 13 magnetic nanoparticle samples

In the present study, 13 magnetic nanoparticles that are various diameters of the same materials Fe_3_O_4 _were precisely prepared by the same way. As shown in Table 1, magnetite average diameters measured by DLS method were almost ten times larger than that of the primary particles measured by TEM method. We considered that some kind of the aggregations of the primary particles occurred. Both average diameters seem to correlate well with each other. However, extremely large SSA was obtained even in the similar DLS diameter, i.e. 74 m^2^/g of SSA in sample D and 190 m^2^/g of SSA in sample M.

**Figure 2:**
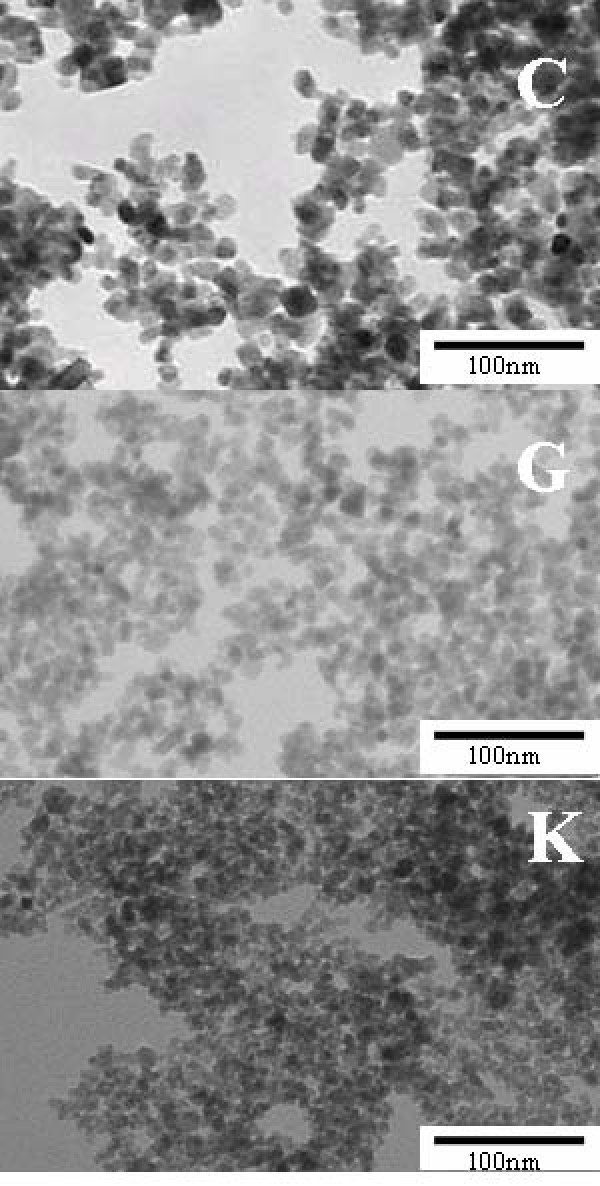
SEM photographs of sample C, G, and K.

For better understanding on physical characteristics of particles, we displayed TEM photographs of particles (Fig. 2). All samples showed even globular or cubic shapes and the rod-shaped particles and larger particles were not included. Polydispersity index of DLS was also listed in Table 1. It was found there was not any particle with extremely wide size distribution.

Saturated magnetization and coercivity were also measured as magnetic properties. These magnetic properties were not correlated with DLS diameter. However, it was found that saturated magnetization was strongly correlated with SSA.

### Temperature profiles by AC magnetic field irradiation

**Figure 3:**
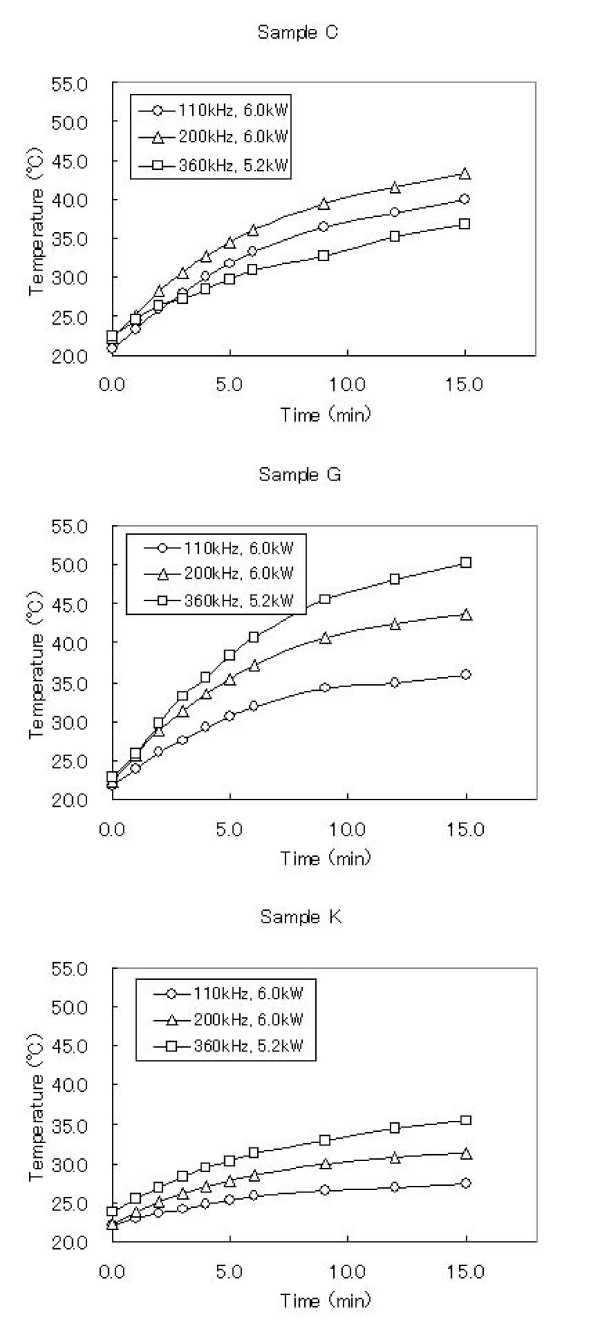
Temperature increasing profiles for several SSA samples and experimental conditions.

**Figure 4:**
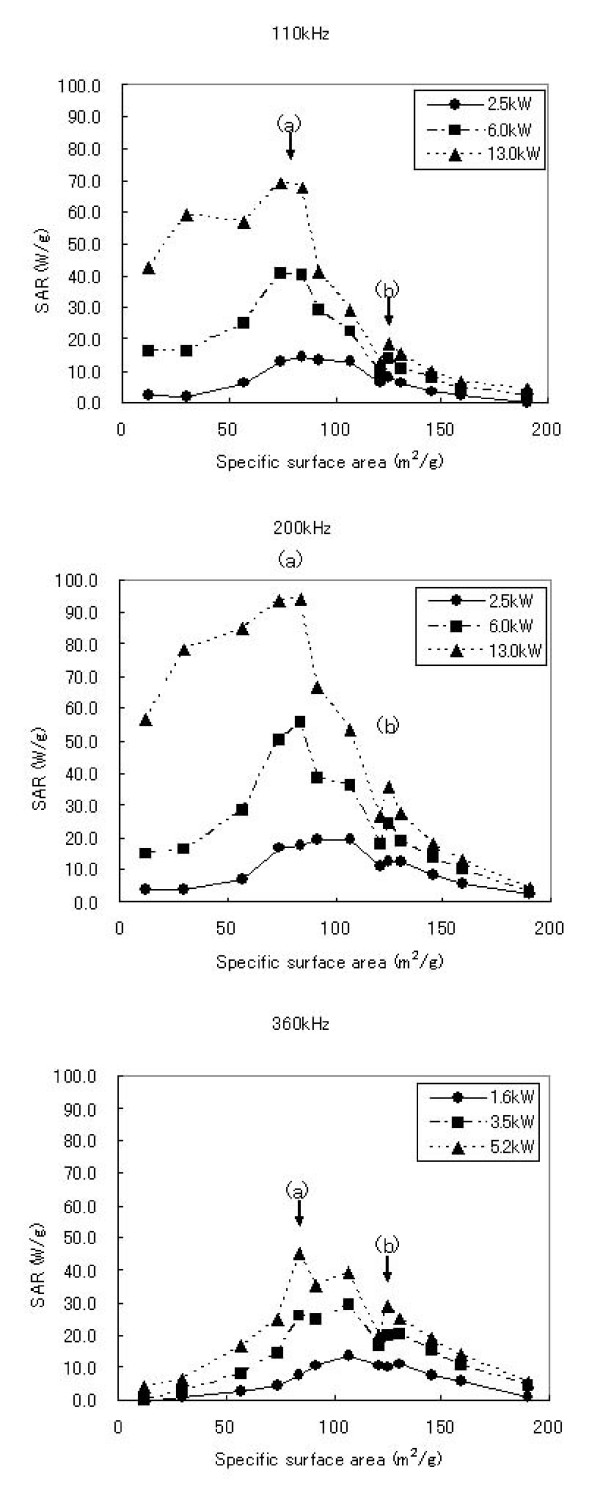
**SAR plots against SSA of the particles.** The experiments performed under the several AMF frequency and power.

**Figure 5:**
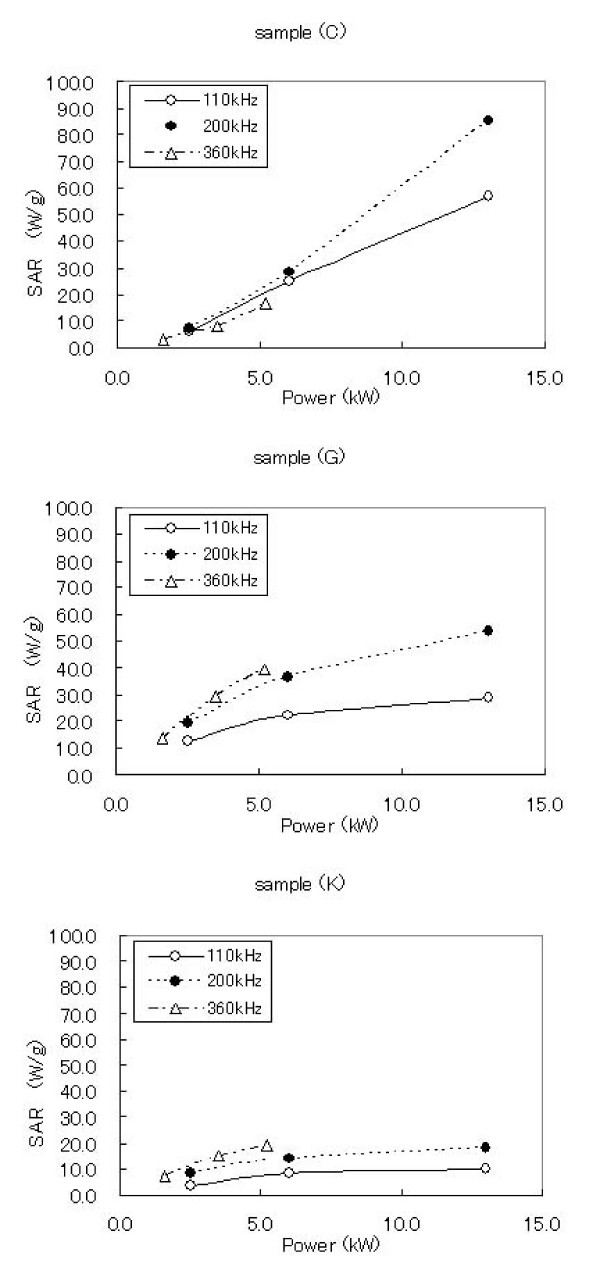
Typical profiles of AMF power dependency to the SAR values represented by sample (C), (G), (K).

Figure 3 shows the typical profiles of the time-dependent temperature curve during the AC magnetic field irradiation. The SAR values (W/g) can be calculated by the following equation [15]:

(1) $$\text{SAR}=\text{C}\frac{\Delta T}{\Delta t}\frac{1}{m_{mag}},$$

where C is the sample-specific heat capacity which is calculated as a mass weighed mean value of magnetite and water. In this study, a heat capacity for magnetite C_mag _was negligible by its low contents in the samples as described below, therefore we use a heat capacity for water C_water _= 4.18 J/gK as the sample's heat capacity. ΔTΔt is the initial slope of the time-dependent temperature curve. As shown in Fig. 3, there are as good as the linear relations in the first rising of the temperature, we use the linear relations in 0–5 minutes intervals for calculating. ΔTΔt m_mag _is the magnetite content per gram of the sample tubes. In this study, the average value of m_mag _was about 5 mg/g. The SAR values of the samples calculated by the equation (1) are shown in Table 3 and plotted against the SSA in Fig. 4. There were two local maximum values of the SAR observed. Also, the SAR was replotted against the power of the AC magnetic field (Fig. 5). The slopes of correlation curve were also calculated in Table 3. As shown in Fig. 5, it was found that heat generation by two processes was found against SSA; In the case of samples with more than 110 m^2^/g of SSA, SAR becomes plateau under excess AC power, but depends on frequency, and SAR depends on AC power in the case of samples with less than 110 m^2^/g of SSA.

**Table 3:** Effects of SSA of the particles on SAR value.

110 kHz				
Samples	SAR (W/g)			Slope
	2.5 kW	6.0 kW	13.1 kW	

A	2.08	16.25	42.60	3.85
B	1.75	16.32	59.30	5.58
C	6.19	25.10	57.08	4.81
D	12.84	40.80	69.21	5.18
E	14.18	40.35	67.82	4.94
F	13.66	29.17	41.16	2.49
G	12.86	22.44	29.03	1.45
H	6.08	8.87	12.77	0.63
I	8.14	13.85	18.16	0.91
J	6.28	10.95	15.37	0.83
K	3.73	8.11	9.78	0.53
L	2.44	4.57	6.54	0.37
M	-0.20	2.27	4.46	0.42

200 kHz				

Samples	SAR (W/g)			Slope
	2.5 kW	6.0 kW	13.0 kW	

A	4.20	14.83	56.63	5.13
B	4.18	16.54	78.53	7.33
C	7.25	28.23	85.06	7.51
D	16.81	50.48	93.54	7.14
E	17.75	55.95	93.72	6.97
F	19.58	38.30	66.60	4.42
G	19.28	36.34	53.72	3.17
H	10.99	18.02	26.46	1.44
I	12.58	24.25	35.84	2.14
J	12.29	19.06	27.43	1.41
K	8.42	13.93	18.12	0.88
L	5.83	9.96	12.83	0.63
M	2.68	3.13	4.45	0.17

360 kHz				

Samples	SAR (W/g)			Slope
	1.6 kW	3.5 kW	5.2 kW	

A	-0.59	-0.10	3.84	1.21
B	1.02	2.59	6.34	1.47
C	2.81	8.03	16.67	3.83
D	4.28	14.72	24.82	5.70
E	7.43	26.10	45.47	10.55
F	10.38	24.79	35.41	6.96
G	13.70	29.42	39.32	7.14
H	10.50	16.79	19.64	2.55
I	10.05	19.80	29.18	5.31
J	10.80	20.06	25.08	3.99
K	7.46	15.30	19.05	3.24
L	5.76	10.56	14.14	2.33
M	0.96	4.22	5.09	1.16

Slopes represent the dependency of the AMF power to the SAR value. The experiments performed under the several AMF frequency and power.

## Discussion

In the present study, 13 magnetic nanoparticles that are various diameters of the same materials Fe_3_O_4 _were precisely prepared by the same way and SAR was measured under various AC frequency and AC power. Such summarized data have been firstly reported by us in the present paper. To the best of our knowledge, it was firstly found that SSA is better index for SAR.

From polydespersity index of DLS listed in Table 1 and TEM photographs, it was found that there was not any particle with extremely wide size distribution. Therefore, it is considerable that there are different degree of aggregates and different packing density of aggregate, because large SSA was obtained even in samples with similar DLS diameter. It is considerable that samples with high packing density behave as a multidomain particle and higher saturated magnetization was obtained.

In Fig. 4, we showed the SARs of the magnetite particles which were measured under variable AMF conditions. There were two local maximum values of the SAR observed when the SARs were plotted against to the SSAs, which were approximately 90 m^2^/g (a) and 120 m^2^/g (b) separately in all the experimental intensities of AC magnetic fields.

It has been reported that the SAR of magnetite particles in an external AC magnetic field can be attributed to two kinds of power loss mechanisms; one is hysteresis loss and others is relaxation loss [16-18]. The grade of these two power losses depends on the particle sizes. The heating due to hysteresis losses are caused by magnetic domain wall displacements under an AC magnetic field. Therefore, it has been reported that the hysteresis loss induced heating needs larger size of magnetic multidomain particles. On the other hand, heating induced by relaxation loss under an AC magnetic field occur to smaller particles that has not domain wall and consists of the single domain structure [18].

The SAR values for the particles below the dividing line which exist around 110 m^2^/g of SSA seemed more susceptible against to the power of AC magnetic field in all frequencies as well. As shown in Fig. 5, it was found that heat generation by two processes was found against SSA. It is strongly suggested that these are happened by clearly different mechanism for heat generation. It is likely that those are relaxation loss and hysteresis loss, respectively, although we could not mention the reason why local maximum is 120 m^2^/g and that was not the smallest SSA in the particles. More profound discussion might be revealed by further experiment. From Table 1, it was found that saturated magnetization was strongly correlated with SSA. In the hysteresis loss, SAR is defined by the area of hysteresis curve. This might be one of the reasons why SSA is better index for SAR, although the reason why saturated magnetization is correlated with SSA still remains to be elucidated.

Since the heating mechanism of the magnetite nanoparticles of different SSA have different attributions from the intensity of AMF, it is considerably needed to optimize the particle SSA for the treatment of MFH. As shown in Fig. 5, the diagram of SAR of the sample K, which has larger SSA, was maintained virtually constant against to the power of AMF in all range of its frequency (Slopes are closed to 0). Therefore for the MFH treatment, when the magnetite particles those have more than 110 m^2^/g of SSA and less than 10 nm of particle diameter are used as the heating mediator, we expect the stable supply of heat could be performed imperviously to the power of AMF.

Comparably, smaller SSA particles generates heat linearly against the strength of the AMF (Slopes in Table 3 are large). That is to say, smaller SSA particles seem to be suited for treating various region of the body part for the MFH treatment because the SAR curve for the smaller SSA particles are adjustable and easily increased linearly by manipulating the power of AMF. In addition the dose of the smaller SSA particles possibly could be hold down when the high-power of AMF are applicable for treatment. It would be also able to heat deep portion of the body part sufficiently by controlling higher dose of the magnetic particles or intensity of the AMF power.

As for the AMF frequency, it should be noted that lower ones within the range of 50 kHz to 100 kHz of AMF are recommended for human therapy depending on the body cross-section and tissue conductivity [7,14]. When we use smaller SSA particles, we could overcome the disadvantage of smaller frequency of the AMF by controlling the intensity of power of the AMF.

Our group is now planning to the application to actual cancer patients. SSA and AC frequency was one of the important criteria for magnetite particle preparation. Actual apparatus for cancer patient was already designed and fabricated, that is AC frequency of 110 kHz. Sample C or D was applicable to actual cancer treatment.

In the present study, we observed collateral evidence that the SAR of the magnetite nanoparticles in an external AMF are induced by two heating mechanisms that depends on the SSA of the particles. The critical change of the SAR value was observed at approximately 110 m^2^/g of the SSA which exists among the 10 nm diameter particles. This is likely due to the structure change of magnetic domain. Additionally, we suggested that heating property of these two mechanisms is defined under the different influences of the frequency and the power of the AC magnetic field.

## Conclusion

In conclusion, we provided the basic data for selection of the magnetite particles for the MFH treatment along with the treating part of the body or purpose of the treatment, and we suggested that for the selection of the particles, SSA could be one of the good criteria.

## Competing interests

The authors declare that they have no competing interests.

## Authors' contributions

All authors contributed equally to the manuscript. All authors have made a significant contribution to this manuscript, and all authors read and approved the final manuscript. 
